# Gastrointestinal Obstruction Secondary to Wilkie's Syndrome: A Case Report of a Rare Clinical Presentation

**DOI:** 10.7759/cureus.96849

**Published:** 2025-11-14

**Authors:** Maria Christou, Antonia Amalia Thanasa, Michail Papamichail, Anastasios Tsokas, Vasileios Vougas

**Affiliations:** 1 1st Department of Surgery and Transplantation, Evangelismos General Hospital, Athens, GRC; 2 Department of Hepatobiliary and Pancreatic Surgery, East Lancashire Hospitals NHS Trust, Blackburn, GBR; 3 Department of Radiology, Evangelismos General Hospital, Athens, GRC

**Keywords:** duodenal obstruction, mesenteric fat loss, nutcracker syndrome, sma syndrome, superior mesenteric artery, upper gi obstruction, wilkie syndrome

## Abstract

Wilkie's syndrome, also known as superior mesenteric artery (SMA) syndrome, is a rare condition characterized by upper gastrointestinal (GI) obstruction caused by compression of the third duodenal portion between the SMA and the abdominal aorta. This report presents a case involving a young female patient who exhibited symptoms of bowel obstruction and a history of chronic malnutrition, loss of appetite, and a depressive disorder. Imaging modalities demonstrated duodenal and gastric dilatation, attributed to a stenosis at the third to fourth portions of the duodenum, due to external compression by the SMA related to significant mesenteric fat loss. After initial conservative management aimed at improving nutritional status, a surgical duodenojejunostomy was performed. The postoperative course was uneventful, and follow-up showed significant weight gain and symptom resolution. SMA syndrome may present as either acute or chronic intestinal obstruction, caused by a variety of risk factors. Differential diagnoses include motility disorders as well as other mechanical causes of duodenal obstruction, and diagnostic modalities include multiple endoscopic and imaging studies. Conservative treatment is preferred as the initial approach, aiming to promote weight gain and restore mesenteric fat, while surgical options are employed upon failure of initial measures. This case highlights that early recognition, appropriate optimization, and timely surgical intervention in high-risk patients can lead to excellent functional outcomes and significant weight recovery.

## Introduction

Superior mesenteric artery (SMA) syndrome, also known as Wilkie’s syndrome or cast syndrome, is a rare yet clinically significant condition occurring in only 0.013%-0.3% of the general population [1-3]. The syndrome is characterized by external compression of the third part of the duodenum due to a reduction in the angle between the SMA and the abdominal aorta, commonly related to the loss of the mesenteric fat pad [4,5]. The left renal vein may also be compressed, resulting in venous congestion, a condition known as nutcracker syndrome [6,7]. SMA syndrome becomes clinically evident when the aortomesenteric angle is abnormally small (normal range: 38°-65°), causing various symptoms, most commonly related to proximal small bowel obstruction [5,8].

The differential diagnosis includes gastroparesis, chronic intestinal pseudo-obstruction, and functional dyspepsia. Excluding primary intestinal motility disorders is essential, as they can present with similar symptoms such as postprandial bloating, nausea, and vomiting. An upper gastrointestinal (GI) series or endoscopy may help exclude intrinsic lesions that cause mechanical obstruction [5,8,9]. Diagnosis is strongly suspected when the aortomesenteric angle is markedly reduced and no other cause of obstruction can be identified.

Patients may initially require conservative management focusing on nutritional support and weight gain [3,8]. Surgical options include bypass procedures such as gastrojejunostomy and duodenojejunostomy. Strong’s procedure involves mobilizing and repositioning the duodenum to the right of the SMA by dividing the ligament of Treitz [10]. This maintains the integrity of the GI tract while eliminating external compression by the SMA or aorta [5,8].

We present the case of a 26-year-old female patient with atypical symptoms of intermittent small bowel obstruction, malnutrition, and weight loss, diagnosed with SMA syndrome and concomitant nutcracker syndrome. After initial conservative management, she was treated surgically with a duodenojejunostomy.

## Case presentation

A 26-year-old female patient presented with chronic malnutrition, loss of appetite, and intermittent partial small bowel obstruction, associated with anxiety and chronic depressive symptoms for a period of 11 years, during which she had not previously sought medical assistance. Her body mass index (BMI) was 16.9 kg/m². Symptoms included dyspepsia, vomiting, abdominal distension, and colicky abdominal pain, progressively worsening over the preceding two to three months. There was no history of abdominal surgery. The initial laboratory evaluations did not suggest a specific pathology, as inflammation markers were within normal ranges, urine analysis was unremarkable, and cancer markers were negative. The patient exhibited mild anemia, with a hematocrit level of 32%. Serum albumin was measured at 3.4 g/dL, serum creatinine was 0.5 mg/dL, and electrolyte levels were within standard ranges, indicating normal renal function.

**Figure 1:**
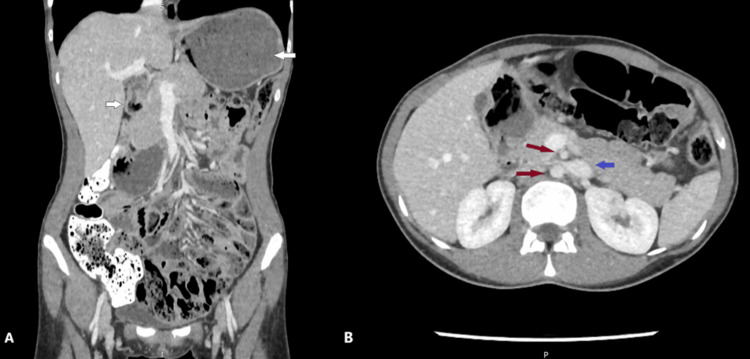
Computed tomography scan (A) Coronal plane. The findings confirm gastric and duodenal dilation, as well as the presence of air-fluid levels proximal to the duodenal stenosis (white arrows). (B) Axial plane. The upper and lower red arrows point to the superior mesenteric artery and abdominal aorta, respectively, and the blue arrow indicates the left renal vein

**Figure 2:**
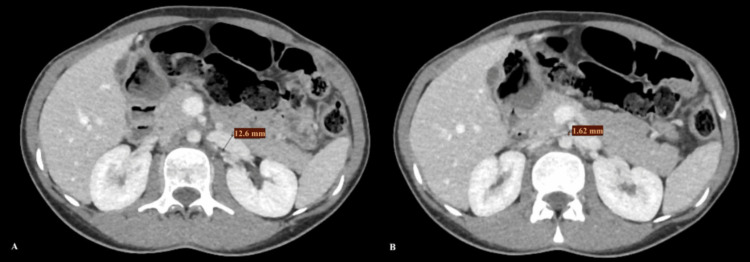
Axial plane intravenous contrast-enhanced computed tomography, demonstrating the compression of the left renal vein by the SMA, with a compression ratio indicating nutcracker syndrome (A) Prestenotic dilation measuring 12.6 mm. (B) Compressed segment measuring 1.62 mm SMA: superior mesenteric artery

**Figure 3:**
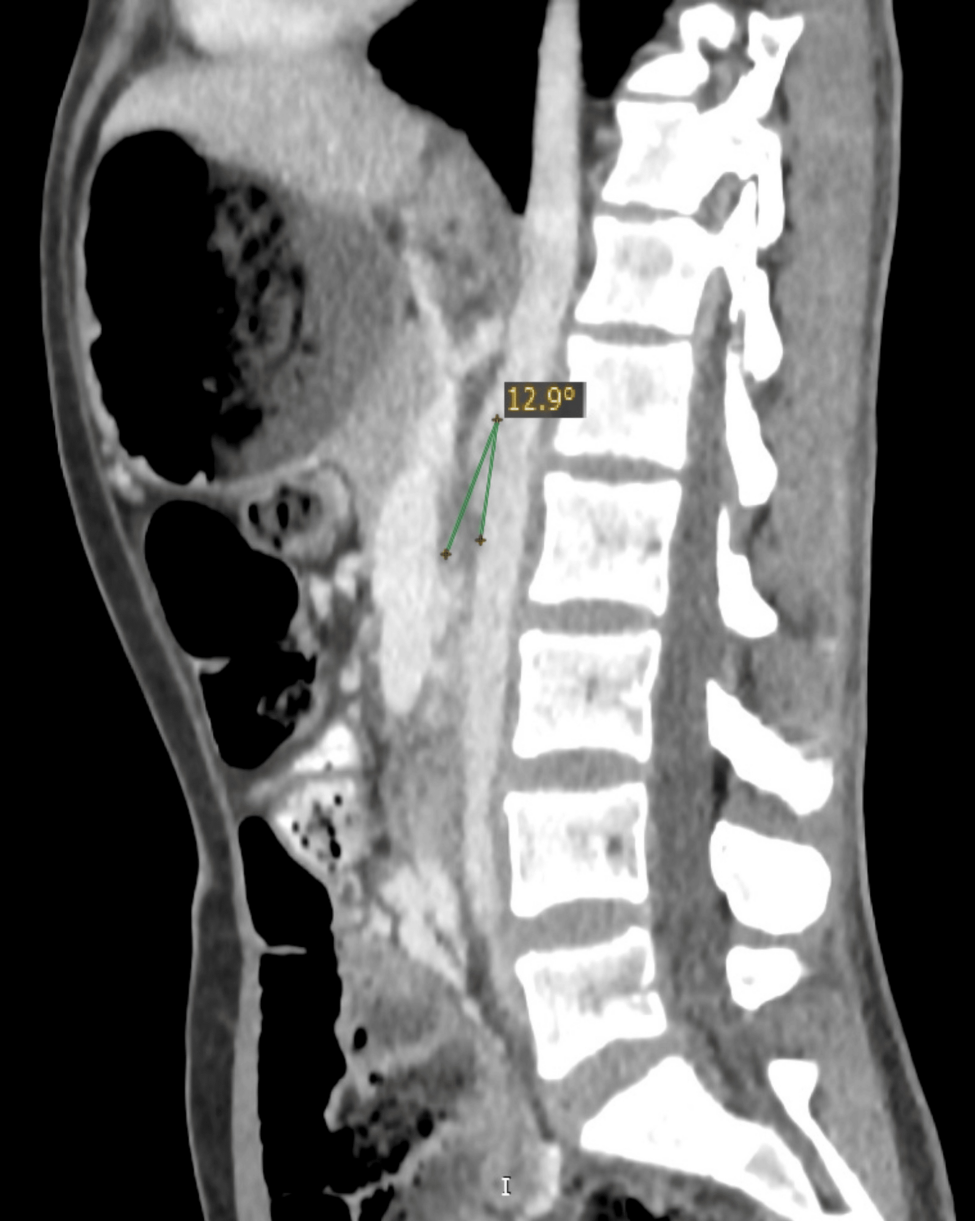
Sagittal plane intravenous contrast-enhanced computed tomography The angle of the aortomesenteric confluence is measured at 12.9°, while normal values of the angle range between 38° and 65°, indicative of SMA syndrome SMA: superior mesenteric artery

Initial imaging, including plain radiographs and computed tomography (CT), showed proximal small bowel dilatation with a transition point at the level of the third to fourth parts of the duodenum (Figure 1), consistent with external compression as the cause of obstruction. Moderate compression of the left renal vein was also noted (Figure 2), and the aortomesenteric angle measured 12.9° (Figure 3). Gastroscopy and endoscopic ultrasonography excluded intraluminal pathology or any other cause of external compression. The diagnosis of Wilkie’s syndrome with associated nutcracker syndrome was therefore strongly suspected.

**Figure 4:**
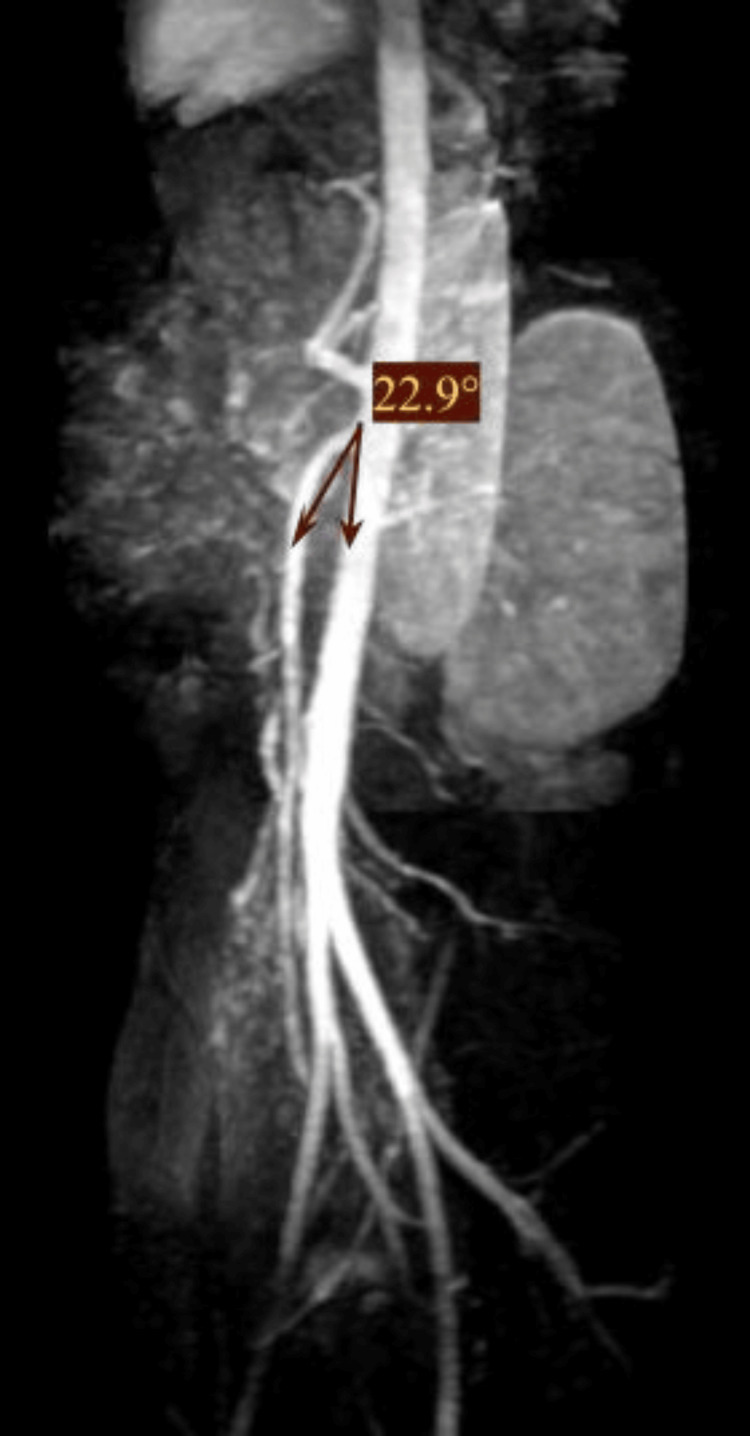
Magnetic resonance angiography The aortomesenteric angle is measured at 22.9°, indicating partial improvement compared to the initial imaging

Conservative management was initiated, focusing on nutritional support (enteral and parenteral feeding) and weight gain. Follow-up magnetic resonance angiography (MRA) after 25 days revealed an increase in the aortomesenteric angle to 22.9°, still below the normal range (Figure 4), with partial clinical improvement. At the patient’s request, a surgical approach was pursued rather than prolonged conservative therapy. A side-to-side duodenojejunostomy was performed. As the patient exhibited no urological symptoms, no intervention was required for the left renal vein compression.

During the operation, the ligament of Treitz was divided, and the proximal jejunum was transected, brought to the right, and resected together with the fourth part of the duodenum. Reconstruction was performed between the third part of the duodenum and the distal small bowel in a side-to-side fashion. The postoperative recovery was uneventful apart from minor wound dehiscence, and the patient was discharged on the ninth postoperative day.

Over the next six months, the patient gained 5 kg, with an increase in BMI to 18.2 kg/m². The only residual symptom was mild gastritis, which was managed with proton pump inhibitors. Later, the patient experienced intermittent episodes of bowel obstruction, which were managed conservatively. Nearly two years after the initial procedure, she was admitted to another hospital with a facial cutaneous infection requiring long-term antibiotics. Subsequently, she developed an acutely distended right colon with partial bowel obstruction, presumed secondary to adhesions or an underlying colitis. A right hemicolectomy with formation of an end ileostomy was performed, while the duodenojejunostomy was preserved and deemed functional. GI continuity was later restored, and the patient remains well with good nutritional status and a BMI of 20.8 kg/m².

## Discussion

SMA syndrome is a rare and often underdiagnosed condition. Compression of the third portion of the duodenum between the abdominal aorta and the SMA [4,5,9] can cause nonspecific GI symptoms, and diagnosis depends on identifying the abnormal aortomesenteric angle.

During embryogenesis [4], paired ventral segmental arteries approach each other in the mesentery to form a median vessel, resulting in a three-vessel visceral arterial system. The SMA arises from the omphalomesenteric artery and a specific segmental artery. Its origin from the aorta is approximately 1 cm below the celiac artery, posterior to the pancreas and splenic vein, and runs inferiorly behind the pancreas at an angle of 38°-65°, crossing anteriorly to the left renal vein. When this angle is significantly reduced, the duodenum becomes compressed between the aorta and the SMA [2].

SMA syndrome typically becomes clinically significant following rapid weight loss, which decreases the fat pad that normally cushions the SMA from the duodenum [5,8]. Risk factors include chronic illness, severe trauma, prolonged immobilization, eating disorders, malabsorption syndromes, spinal deformities or surgery (e.g., scoliosis correction), burns, bariatric procedures, and substance abuse [1,3,5]. The estimated incidence ranges from 0.013% to 0.78% [1-3], with the condition most commonly affecting adolescents and young adults, and showing a female predominance (female-to-male ratio of up to 4:1) [1,2].

Nutcracker syndrome, often associated with SMA syndrome, results from compression of the left renal vein between the SMA and the aorta, leading to impaired renal venous drainage [6,7]. Symptoms include hematuria, flank or pelvic pain, anemia, proteinuria, left-sided varicocele in men, and dysmenorrhea in women [6]. Depending on severity, management options include stenting, renal vein transposition, or autotransplantation [6,7]. Combined duodenal and vascular surgery may be necessary in complex cases with significant symptoms [7].

Diagnosis of SMA syndrome is suspected when the clinical presentation correlates with a reduced aortomesenteric angle and no evidence of intestinal dysmotility or other mechanical obstruction [3]. Nonspecific symptoms include abdominal pain, bloating, early satiety, nausea, vomiting, and weight loss [5]. In severe cases, patients may develop complete obstruction and an inability to tolerate oral intake. The frequency of the symptoms underscores the clinical importance of being aware of this uncommon condition, particularly given that the initial differential diagnosis typically involves more prevalent diseases. Imaging plays a crucial role in diagnosis; abdominal radiographs may show small bowel dilatation or air-fluid levels, and contrast-enhanced CT can confirm duodenal compression and measure the aortomesenteric angle [8,9,11]. Additional studies, such as MRA, upper GI series, motility studies, and endoscopy, may help further delineate anatomy and exclude alternative causes [1,9,11].

In this case, the chronicity of symptoms and long-standing malnutrition in a young woman (possibly with undiagnosed anorexia nervosa), along with radiological evidence of nutcracker syndrome, strongly supported the diagnosis of SMA syndrome in the absence of other causes, such as a tumor or prior abdominal surgery.

Conservative treatment options include proximal decompression, nutritional support (enteral or parenteral feeding), correction of metabolic abnormalities, and physical therapy to promote weight gain and restore the aortomesenteric fat pad [3,8]. Psychiatric assessment is recommended if an eating disorder is suspected. Surgical intervention is indicated when symptoms persist despite conservative measures [1]. Preoperative optimization is crucial for correcting metabolic imbalances, enhancing muscle mass, improving healing capacity, and reducing postoperative complications.

Surgical options include duodenojejunostomy, gastrojejunostomy, and Strong’s procedure [1,2,8]. Duodenojejunostomy is the most widely performed and effective, offering durable symptom relief and a low recurrence rate. Gastrojejunostomy is another option, but it may lead to long-term complications such as bile reflux and ulceration [1,2]. Strong’s procedure involves dividing the ligament of Treitz and mobilizing the duodenum to the right of the SMA, avoiding bypass of the intestinal tract; it is mainly used in children or when duodenostomy is contraindicated [1,8]. Laparoscopic approaches have been increasingly reported, offering shorter recovery and lower morbidity [12-14]. Studies have shown high rates of symptom resolution and weight gain, with one study reporting sustained improvement and a BMI increase after a median four-year follow-up following duodenojejunostomy [15].

In our case, a duodenojejunostomy was the operation of choice, as it is a well-established and effective alternative that minimizes the aforementioned side effects associated with gastrojejunostomy. The procedure was performed following mobilization and resection of the distal duodenum and proximal jejunum, rather than simple bypass. Resection of the affected bowel segment may reduce the risk of stasis, bacterial overgrowth, or functional obstruction in the bypassed duodenum, while preserving normal flow of biliary and pancreatic secretions.

While surgical intervention often leads to early symptom relief, long-term follow-up is necessary. In studies of laparoscopic duodenojejunostomy, initial improvement occurred in most patients, but 21%-66% developed recurrent symptoms over time, including dysmotility, gastroparesis, anastomotic stricture, or dumping syndrome [12,13]. Another study reported that 28% of patients required further major interventions for persistent motility disorders despite initial improvement, including median arcuate ligament release, colectomy, or even intestinal transplantation [14].

Our patient later developed severe large bowel dilatation, likely due to colitis or a closed-loop syndrome, requiring surgical intervention. Despite this, more than two years after her initial surgery, she had achieved significant weight gain and remains in good condition. Long-term follow-up will be necessary to monitor for potential future dysmotility complications.

## Conclusions

This case highlights the diagnostic and therapeutic challenges of SMA syndrome as a rare cause of intestinal obstruction, particularly in young patients with atypical symptoms and complex psychosocial backgrounds. It is crucial to exclude motility disorders when considering SMA syndrome. Early recognition and timely surgical intervention, following appropriate conservative management and optimization, can lead to significant improvement and better quality of life.
